# Anabolic Androgen Abuse-Associated Cerebral Venous Sinus Thrombosis in a Young Adult: A Case Report

**DOI:** 10.7759/cureus.102234

**Published:** 2026-01-24

**Authors:** Anas E Ahmed, Albaraa H Sahli, Mishari L Alenezi, Obaeda A Jamor, Mohammed A Alsubaie

**Affiliations:** 1 Community Medicine, Jazan University, Jazan, SAU; 2 College of Medicine, Abu-Hajar Primary Health Care Center, Jazan, SAU; 3 College of Medicine, University of Hail, Hail, SAU; 4 Medicine and Surgery, Batterjee Medical College, Jeddah, SAU; 5 College of Medicine, King Faisal University, Al-Ahsa, SAU

**Keywords:** anabolic androgenic steroids, androgen abuse, cerebral venous sinus thrombosis, hypercoagulable state, secondary polycythemia, seizure, venous thrombosis, young adult

## Abstract

Cerebral venous sinus thrombosis (CVST) is a rare and potentially life-threatening cause of stroke that predominantly affects young adults and often presents with nonspecific symptoms, leading to diagnostic delay. We report the case of a 29-year-old man with no prior medical comorbidities who presented with a progressively worsening headache and new-onset generalized tonic-clonic seizure. Detailed history revealed long-term abuse of anabolic androgenic steroids for bodybuilding purposes. Clinical examination demonstrated papilledema, and laboratory investigations were notable for secondary polycythemia, while an extensive thrombophilia workup was unremarkable. Neuroimaging with computed tomography and magnetic resonance venography confirmed thrombosis of the superior sagittal and right transverse sinuses with associated venous infarction. The patient was managed with therapeutic anticoagulation, antiepileptic therapy, and measures to control intracranial pressure, alongside immediate cessation of androgen use. His clinical course was favorable, with symptomatic improvement, partial radiological recanalization, and complete neurological recovery at follow-up. This case highlights androgen abuse as a rare but important reversible risk factor for CVST and underscores the importance of thorough substance use history taking, early diagnosis, and prompt anticoagulation to optimize outcomes.

## Introduction

Cerebral venous sinus thrombosis (CVST) is a rare form of cerebrovascular disease, representing approximately 0.5%-1% of all strokes, and predominantly affects young and middle-aged adults [1,2]. Unlike arterial stroke, CVST exhibits a wide spectrum of clinical manifestations, ranging from isolated headache to seizures, focal neurological deficits, altered consciousness, and intracranial hemorrhage [1,2]. This variability often leads to diagnostic delays and misdiagnosis, particularly in patients without obvious predisposing conditions [2,3]. Early recognition is critical, as timely anticoagulation can significantly reduce morbidity and mortality [1-3].

The pathogenesis of CVST is multifactorial, involving both inherited and acquired prothrombotic risk factors [3,4]. Commonly recognized causes include pregnancy and the puerperium, oral contraceptive use, systemic infections, malignancy, autoimmune disorders, dehydration, trauma, and inherited thrombophilias [1,3]. However, in a substantial proportion of cases, no clear etiology is identified after extensive investigation [1,2]. In young male patients, the absence of traditional risk factors should prompt consideration of less common causes, including substance-related hypercoagulable states.

Anabolic androgenic steroids are synthetic derivatives of testosterone that are widely misused for esthetic and performance-enhancing purposes [4,5]. Although often perceived as relatively safe by users, androgens have been associated with significant cardiovascular and thrombotic complications [1,3]. Proposed mechanisms include androgen-induced polycythemia, increased platelet aggregation, alterations in coagulation and fibrinolytic pathways, and endothelial dysfunction, all of which contribute to a hypercoagulable state [2,3,5]. While venous thromboembolism related to androgen abuse has been increasingly reported, CVST remains a rare and underrecognized manifestation. Documenting such cases is important to raise clinical awareness, encourage detailed substance use history taking, and highlight the potentially serious neurological consequences of anabolic steroid misuse.

## Case presentation

A 29-year-old man was admitted to the emergency department with a five-day history of progressively worsening headache, described as diffuse, severe, and pressure-like, with a predominance in the occipital region. The headache was refractory to over-the-counter analgesics and was associated with nausea, intermittent vomiting, photophobia, and transient visual blurring. Twenty-four hours prior to presentation, he developed a generalized tonic-clonic seizure lasting approximately one minute, followed by postictal confusion, which prompted urgent medical evaluation. There was no prior history of seizures, migraine, head trauma, fever, recent infection, dehydration, prolonged immobilization, or recent surgery. He denied focal weakness, speech disturbance, or loss of consciousness apart from the seizure episode.

The patient had no known chronic medical illnesses and was not taking prescribed medications. However, on detailed history taking, he disclosed self-administered anabolic androgenic steroid use for bodybuilding purposes for approximately three years. His regimen included intramuscular testosterone enanthate and oral methandrostenolone, used in cyclical high doses without medical supervision. He denied using other illicit drugs, including cocaine or amphetamines. There was no personal or family history of thromboembolic disease, autoimmune disorders, malignancy, or known inherited thrombophilia. He was a nonsmoker and consumed alcohol occasionally. Review of systems was otherwise unremarkable.

On examination, the patient was alert but in significant distress due to headache. Vital signs revealed blood pressure of 148/92 mmHg, heart rate of 96 beats per minute, respiratory rate of 18 breaths per minute, temperature of 36.8°C, and oxygen saturation of 98% in room air. Neurological examination demonstrated bilateral papilledema on fundoscopic examination, suggesting raised intracranial pressure. Cranial nerve examination was otherwise intact. Motor strength was 5/5 in all extremities, with normal tone and reflexes. Sensory examination and cerebellar testing were unremarkable. There were no signs of meningism. Cardiovascular, respiratory, and abdominal examinations were within normal limits, and no peripheral edema or calf tenderness was noted.

Initial laboratory investigations revealed hemoglobin of 18.2 g/dL (reference range: 13.5-17.5 g/dL for men; 12.0-15.5 g/dL for women) and hematocrit of 54% (reference range: 41%-50% for men; 36%-48% for women), consistent with secondary polycythemia. White blood cell count and platelet count were within normal limits. Serum electrolytes, renal function, and liver function tests were largely normal, apart from mildly elevated alanine aminotransferase. The coagulation profile showed normal prothrombin time and activated partial thromboplastin time. D-dimer levels were elevated. Inflammatory markers, including C-reactive protein and erythrocyte sedimentation rate, were not significantly raised. A comprehensive thrombophilia workup was performed, including protein C, protein S, antithrombin III levels, factor V Leiden mutation, prothrombin gene mutation, antiphospholipid antibodies, and homocysteine levels, all of which were within normal limits or negative. Viral serology, including HIV and hepatitis panel, was negative.

**Figure 1:**
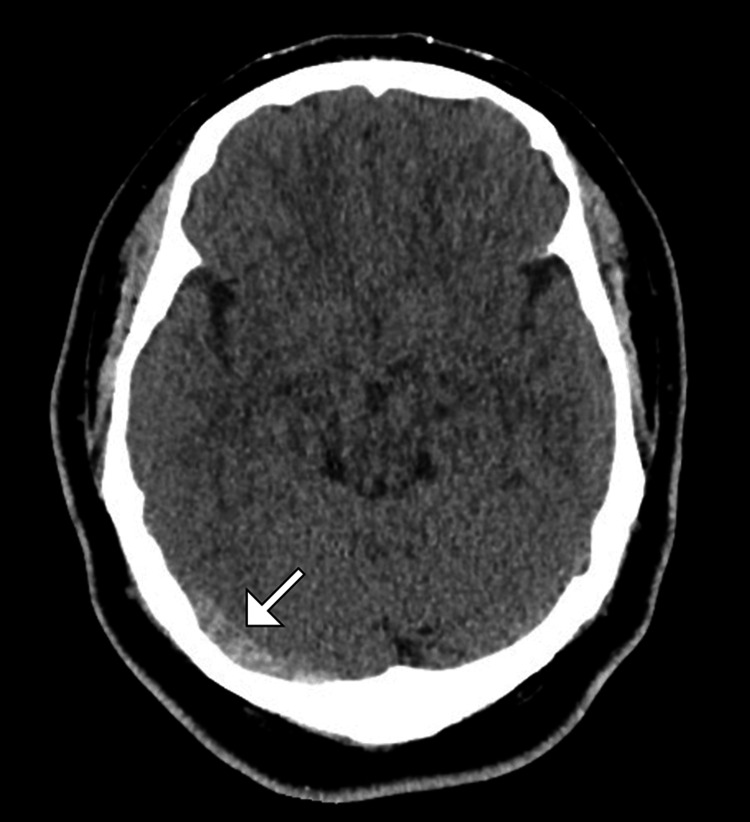
Axial non-contrast brain computed tomography image showing hyperdensity (arrow) along the transverse sinus

**Figure 2:**
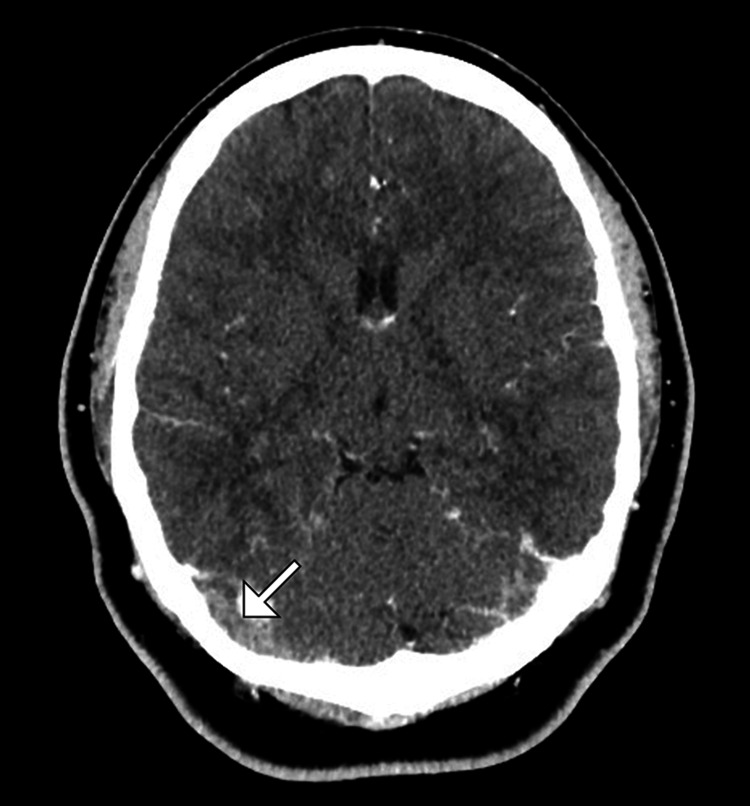
Axial computed tomography venogram showing a filling defect (arrow) along the right transverse sinus, confirming the presence of thrombosis

Non-contrast computed tomography of the brain demonstrated hyperdensity along the right transverse sinus (Figure 1 and Figure 2). There was no evidence of arterial infarction, space-occupying lesion, or intracranial infection.

The differential diagnosis considered included subarachnoid hemorrhage, intracranial mass lesion, idiopathic intracranial hypertension, central nervous system infection, arterial ischemic stroke, and reversible cerebral vasoconstriction syndrome. These were systematically excluded based on clinical presentation, laboratory findings, and neuroimaging. Given the absence of other prothrombotic conditions and the presence of long-term high-dose androgen abuse with secondary polycythemia, the CVST was attributed to androgen-induced hypercoagulability.

The patient was admitted to the neurological intensive care unit and initiated on therapeutic anticoagulation with low-molecular-weight heparin, despite the presence of a small hemorrhagic component, in accordance with current guidelines. Levetiracetam was started for seizure prophylaxis, and acetazolamide was administered to manage raised intracranial pressure. Androgen use was immediately discontinued, and the patient received counseling regarding the risks of anabolic steroid abuse. Serial neurological assessments showed gradual improvement in headache severity, and no further seizures occurred during hospitalization.

During his hospital course, repeat imaging performed on day 7 demonstrated partial recanalization of the affected sinuses and reduction in cerebral edema. He was transitioned to oral anticoagulation with warfarin, with a planned duration of at least 6-12 months, and discharged home in stable condition after 10 days of hospitalization. At the three-month follow-up, the patient reported complete resolution of headaches and no recurrent seizures.

## Discussion

CVST is an uncommon but important cause of stroke in young adults, characterized by heterogeneous clinical manifestations and diverse etiologies [1-4]. The present case highlights androgen abuse as a rare but clinically significant risk factor for CVST, particularly in young male patients without traditional prothrombotic conditions. The patient’s presentation with progressive headache and new-onset seizure is consistent with the most frequently reported symptoms of CVST, reflecting elevated intracranial pressure and cortical irritation secondary to impaired venous drainage [2,4,5]. This case reinforces the need for a high index of suspicion for CVST when evaluating atypical headache and seizure presentations in otherwise healthy individuals.

The association between anabolic androgenic steroids and thrombotic events has been increasingly recognized, although CVST remains infrequently reported in this context [1,4,5]. Several pathophysiological mechanisms may explain the prothrombotic effects of androgens. Androgens stimulate erythropoiesis, often resulting in secondary polycythemia, as observed in this patient, which increases blood viscosity and predisposes to venous stasis [6,7]. Additionally, anabolic steroids have been shown to enhance platelet aggregation, alter levels of coagulation factors, suppress fibrinolysis, and induce endothelial dysfunction [2-4]. These effects collectively promote a hypercoagulable state, increasing the risk of both arterial and venous thrombosis [3,4]. In the absence of inherited thrombophilia, systemic inflammatory disease, malignancy, or other recognized risk factors, androgen abuse was considered the most plausible etiological factor in this case.

Diagnosing CVST can be challenging due to its variable presentation and the limitations of initial imaging modalities [3,5]. Non-contrast computed tomography may be normal or demonstrate subtle findings, whereas magnetic resonance imaging combined with venography remains the diagnostic gold standard [1,3,5]. In this patient, early neuroimaging was crucial in establishing the diagnosis and preventing further neurological deterioration. The differential diagnosis included subarachnoid hemorrhage, intracranial mass lesion, idiopathic intracranial hypertension, and arterial ischemic stroke, all of which were excluded based on imaging and laboratory findings [3,5]. This emphasizes the importance of comprehensive neuroimaging in patients with unexplained headache, seizures, or signs of raised intracranial pressure.

Therapeutic anticoagulation remains the cornerstone of CVST management, even in the presence of hemorrhagic venous infarction. The favorable clinical and radiological response observed in this case aligns with existing evidence demonstrating improved outcomes with early anticoagulation [1-5]. Adjunctive therapies, including antiepileptic medication and measures to reduce intracranial pressure, are tailored to individual clinical features [4,5,7]. Importantly, identification and elimination of the underlying precipitating factor are essential to prevent recurrence. In this context, cessation of androgen abuse and patient counseling played a critical role in long-term management [2,3,6].

This case underscores several important clinical implications. First, a detailed substance use history, including non-prescribed performance-enhancing drugs, should be routinely obtained in young patients presenting with thrombotic events [3,5]. Second, clinicians should be aware of androgen abuse as a potentially reversible cause of CVST [1,5,6]. Finally, increased reporting of such cases is necessary to enhance understanding of this rare association, inform risk stratification, and guide preventive strategies. As anabolic steroid misuse continues to rise globally, heightened awareness of its neurological complications is essential to reduce preventable morbidity in this population.

## Conclusions

In conclusion, this case illustrates CVST as a rare but serious neurological complication of anabolic androgen abuse in a young man without conventional thrombotic risk factors. It emphasizes the importance of maintaining a high index of suspicion for CVST in patients presenting with atypical headache and seizures, as well as the critical role of thorough substance use history taking in identifying uncommon yet reversible etiologies. Early diagnosis, prompt anticoagulation, and cessation of androgen use were central to the favorable outcome observed, underscoring the need for increased clinical awareness and reporting of androgen-related thrombotic events to improve prevention, recognition, and management strategies.
